# Development and Characterization of Methyl-Anthranilate-Loaded Silver Nanoparticles: A Phytocosmetic Sunscreen Gel for UV Protection

**DOI:** 10.3390/pharmaceutics15051434

**Published:** 2023-05-08

**Authors:** Mohammed Ghazwani, Umme Hani, Mohammed H. Alqarni, Aftab Alam

**Affiliations:** 1 Department of Pharmaceutics, College of Pharmacy, King Khalid University, P.O. Box 1882, Abha 61441, Saudi Arabia; 2 Department of Pharmacognosy, College of Pharmacy, Prince Sattam Bin Abdulaziz University, Al Kharj 11942, Saudi Arabia

**Keywords:** methyl anthranilate, silver nanoparticles, dermatokinetic, antioxidant, sunscreen gel

## Abstract

Methyl anthranilate (MA) is a naturally derived compound commonly used in cosmetic products, such as skin care products, fine perfumes, etc. The goal of this research was to develop a UV-protective sunscreen gel using methyl-anthranilate-loaded silver nanoparticles (MA-AgNPs). The microwave approach was used to develop the MA-AgNPs, which were then optimized using Box–Behnken Design (BBD). Particle size (Y1) and absorbance (Y2) were chosen as the response variables, while AgNO_3_ (X1), methyl anthranilate concentration (X2), and microwave power (X3) were chosen as the independent variables. Additionally, the prepared AgNPs were approximated for investigations on in vitro active ingredient release, dermatokinetics, and confocal laser scanning microscopy (CLSM). The study’s findings showed that the optimal MA-loaded AgNPs formulation had a particle size, polydispersity index, zeta potential, and percentage entrapment efficiency (EE) of 200 nm, 0.296 mV, −25.34 mV, and 87.88%, respectively. The image from transmission electron microscopy (TEM) demonstrated the spherical shape of the nanoparticles. According to an in vitro investigation on active ingredient release, MA-AgNPs and MA suspension released the active ingredient at rates of 81.83% and 41.62%, respectively. The developed MA-AgNPs formulation was converted into a gel by using Carbopol 934 as a gelling agent. The spreadability and extrudability of MA-AgNPs gel were found to be 16.20 and 15.190, respectively, demonstrating that the gel may spread very easily across the skin’s surface. The MA-AgNPs formulation demonstrated improved antioxidant activity in comparison to pure MA. The MA-AgNPs sunscreen gel formulation displayed non-Newtonian pseudoplastic behaviour, which is typical of skin-care products, and was found to be stable during the stability studies. The sun protection factor (SPF) value of MA-AgNPG was found to be 35.75. In contrast to the hydroalcoholic Rhodamine B solution (5.0 µm), the CLSM of rat skin treated with the Rhodamine B-loaded AgNPs formulation showed a deeper penetration of 35.0 µm, indicating the AgNPs formulation was able to pass the barrier and reach the skin’s deeper layers for more efficient delivery of the active ingredient. This can help with skin conditions where deeper penetration is necessary for efficacy. Overall, the results indicated that the BBD-optimized MA-AgNPs provided some of the most important benefits over conventional MA formulations for the topical delivery of methyl anthranilate.

## 1. Introduction

The ultraviolet (UV) rays of sunlight, which can result in skin cancer, are one of the most significant causes of pathological conditions of the skin. A unique wavelength of sunlight known as UV radiation is broken down into three ranges: UVC (200–290 nm), UVB (290–320 nm), and UVA (320–400 nm) [1]. Due to their ability to destroy skin collagen, produce free radicals, and prevent skin regeneration processes, types A and B can result in sunburn, wrinkles, and skin cancer [2]. The damage induced by UVC is more severe than that of UVB and UVA because shorter UV wavelengths do more harm to the human body. However, because UVC is generally absorbed by the ozone layer in the atmosphere, UVB is the predominant UV radiation causing skin redness, sunburn, the production of reactive oxygen species (ROS), DNA damage, and skin cell death [3]. The UV Index (UVI) was established by the World Health Organization (WHO) to forecast, report, and measure the amount of UV radiation [4]. The damages due to UVR on a variety of skin colors are markedly different among races and populations. The diseases or pathological skin conditions that are caused by UV radiation are inflammatory skin diseases (UVR can be both the cause of and cure for this condition), psoriasis, atopic dermatitis, vitiligo, skin cancer, acne, local infections, sunburn, premature ageing, and other diseases such as solar keratoses, cortical cataracts, pterygium, and herpes labialis [5,6,7]. The UVA radiation effectively enters the dermis and the lower subcutaneous fat tissue, UVB radiation only penetrates the epidermis and the top layer of the dermis. It is also obvious that if only a tiny portion of the body is exposed to radiation as opposed to the entire body, various reactions may take place. Chronic exposure may result in photoadaptation, which reduces future responses to equal doses of UVR, and photoprotection, which prevents the expected reactions to a single high dose of UVR from occurring. Tanning, epidermal hyperplasia, and stratum corneum hyperkeratosis are likely the underlying causes [8]. Sunscreen lotions, creams, and gels were developed in order to shield skin from the damaging effects of the sun [3]. There are many different qualities that a good sunscreen should have. It needs to have a strong, broad absorption band in the UV portion of the spectrum, and it needs to quickly revert to its ground state after being exposed to UV-radiation without developing any hazardous intermediates. In addition to being photostable across an appropriate exposure range, the molecule should also be nontoxic and non-phototoxic [9].

Licorice root extract (*Glycyrrhiza glabra*) and jasmine flowers (*Jasminum officinale*) are the sources of methyl anthranilate (MA) [10]. Skin cancer, antimicrobials, and other skin disorders are among the illnesses, conditions, and symptoms that methyl anthranilate is used to treat, control, prevent, and improve [11]. Methyl anthranilate, a UV filter, has a maximum absorbance of 288 and 325 nm and is a promising UV-blocking agent since it can reflect and absorb a broad spectrum of UV radiation (200–400 nm) [12]. MA is typically used in conjunction with other UV filters and has been given FDA approval by the United States [13]. In order to avoid photocarcinogenesis, it is also utilised as an ultraviolet absorber in many sunscreen products. Additionally, MA was discovered to exhibit photoprotective properties [14,15]. The intramolecular hydrogen bonding facilitated by the NH2 group’s ortho position with respect to the ester substituent, the anthranilate class of sunscreens is regarded as photostable [16]. Octyl methoxycinnamate is the most widely used UV-B sunscreen in contemporary formulations. MA is described as “a good sunscreen to add to formulations containing octylp-methoxycinnamate to both boost the SPF and give protection in the UV-A area” [13]. The use of MA as the primary active component in sunscreen is restricted despite its photoprotective, antioxidant, and UVA filter characteristics. MA is non-hydrolyzable but is susceptible to photoreaction when exposed to UV light [17], which could lower its effectiveness and acceptability as a photoprotection agent [18].

Innovative and more potent formulations are still required to meet these demands since UV filters must be kept on the highest skin regions. To reduce the danger of toxicity and/or negative side effects, it is necessary to guarantee there is no systemic absorption [19]. To overcome the drawbacks associated with conventional topical formulations such as low penetration and retention in the skin’s outer layers, instability of the active components, poor water resistance, and/or ROS production by inorganic filters [20]. It is anticipated that the use of nanosized carriers in active ingredient delivery will improve active ingredient specificity, efficacy and, as a result, minimise side effects while lowering dosage. Site-specific skin targeting is made possible by nanotechnology-driven active ingredient delivery systems, which may lead to higher active ingredient retention at the target site [21]. Cosmetics, dermatology, and other biological applications are just a few of the areas where nanotechnology has recently witnessed significant advancements and broad use. Nanoparticles (NPs) can range in size below 200 nm. Nanoparticles (NPs), which are too small for the naked eye to detect, have the potential to radically alter surfaces and structures by introducing cleaner, lighter, stronger, and “smarter” physical and chemical qualities [22]. The cosmetic industry was one of the first to adopt nanotechnology because of its considerable contribution to better skin penetration, stability, and component release through the skin barrier, which results in greater cosmetic effects [23]. Numerous cosmeceutical goods, including sunscreen, moisturizing and whitening creams, face cleansers, shampoos for hair repair, and anti-ageing products, contain nanotechnology [24]. Antioxidant nanoparticle use in sunscreen formulations has attracted a lot of attention in recent years. Titanium oxide and zinc oxide are the two most prevalent varieties of man-made nanomaterials. However, additional nanosized materials have started to be employed in cosmetic applications, including metals such as gold and silver, metal oxides, liposomes, nanocapsules, cubosomes, dendrimers, niosomes, and solid lipid nanoparticles [25]. Since the late 1990s, TiO_2_ and ZnO nanoparticles (NPs) have been common constituents in commercial sunscreens [26]. To prevent sunburn, experts advise applying sunscreen to block UV rays and limiting your exposure to the sun. Sunscreen formulation efficacy is measured by its capacity to shield skin from the sun’s ultraviolet (UV) rays, and its performance is determined by its sun protection factor (SPF) [27]. TiO_2_ and ZnO can protect against and lessen UV irradiation-induced damage by reflection, scattering, and absorption, respectively [28,29]. However, systemic absorption and photocatalytic ROS generation have raised more and more health issues [30]. Nanosized TiO_2_ that has been contaminated with anatase crystals can cause photocatalysis and cellular damage [31]. Therefore, a reliable and efficient TiO_2_ and ZnO replacement is needed.

Since ancient times, silver has been employed as a bactericidal agent for hygiene and medical purposes [32]. Silver nanoparticles (AgNPs) are not harmful to cells grown in vitro [33] and are presently extensively utilized in nanomedicine for treating and caring for burns, trauma, acne, and diabetic wounds, as well as in catheters, contraceptive devices, dental silver amalgams, and water purifying equipment [34,35,36]. As a result, AgNPs constitute a rapidly expanding market for products based on nanotechnology [37]. Following a sunburn, bacterial infections are a worry. Due to their relatively large surface area, which allows for better interaction with microbes, silver nanoparticles demonstrate effective antibacterial properties and also possess antioxidant characteristics by scavenging relative oxidative stress [38,39]. AgNPs can stabilise MA and block its photoreaction to UV light. This is due to the fact that AgNPs can serve as a physical barrier between MA and UV radiation, limiting the possibility of photoreaction by preventing direct contact or by reducing the intensity of UV radiation [40]. As a result, AgNPs might be especially well suited as carriers in sunscreen. AgNPs-related research has mostly employed in vitro models to conduct feasibility analyses [41,42]. AgNPs are metal nanoparticles that display the surface plasmon resonance (SPR) feature. AgNPs are extremely effective in both light absorption and scattering. Conduction electrons collectively oscillate when metal atoms on a metal surface are excited by photons of a particular wavelength. SPR-based absorption spectroscopy is dependent on the metal surface’s dimensions, composition, and reactivity to surface modifications [43]. We hypothesised that, in light of the information that AgNPs showed UV blocking properties, ROS regulation, and maintained antibacterial characteristics, AgNPs could be a promising carrier for methyl anthranilate by enhancing UV-blocking activity and SPF values.

Since nanomaterials have the potential for active ingredient delivery, the current work intends to develop MA-loaded silver nanoparticle sunscreen gel to provide a synergistic effect for UV protection, as both MA and silver nanoparticles have a shielding effect against UV radiation. Our composition, we believed, had a number of advantages, including great spreadability, improved penetration, better retention time, and a synergistic impact because silver nanoparticles have a larger surface area to cover the skin. On the other hand, silver nanoparticles have strong anti-bacterial activity, which can protect the skin after sunburn, as bacterial infection is one of the frequent occurrences following UV-radiation-induced skin damage. The Box–Behnken design was employed for optimization. To achieve this aim, the influence of various parameters, i.e., silver nitrate concentration, amount of MA, and power of microwave on the size of silver particles, and absorbance, was investigated. The optimized AgNPs were then characterized; furthermore, a topical gel was formulated, and its characterization was performed. However, it should be noted that extensive testing is necessary before approving any novel formulation for human application. Therefore, additional pre-clinical, clinical, and dermatologically tested studies are needed to determine whether or not these formulations can be brought to market with a lower risk/benefit ratio than the current formulation.

## 2. Materials and Methods

### 2.1. Materials

Methyl anthranilate was procured from Sigma Aldrich (Mumbai, India), and silver nitrate in the form of solid crystals was purchased from Thomas Baker (Mumbai, India). Methanol was of HPLC grade and purchased from S.D. Fine Chemicals Ltd. (Mumbai, India). Triethanolamine and polyethylene glycol (PEG) were obtained from S.D. Fine Chemicals Ltd. (Mumbai, India). Carbopol-934 was purchased from B.S. Goodrich, Pleveland. Other agents used in experimentation were of analytical grade.

### 2.2. Development of Silver Nanoparticles

The MA solution was mixed with a 1 mM silver nitrate solution while being continuously stirred at room temperature to generate the MA-AgNPs. Then, at a predetermined frequency of 2.45 GHz and a temperature of 90 °C, the solution was microwave irradiated. The color of the solution changed from clear to dark brown. This shift in color signalled the formation of silver nanoparticles, which was later confirmed by UV spectroscopy, which revealed lambda max to be greater than 400 nm. Following centrifugation, silver nanoparticles were cleaned with distilled water and acetone to get rid of any water-soluble particles. The characterization of the powdered nanoparticles served to verify their production [36].

### 2.3. Optimization of Silver Nanoparticles

Silver nanoparticles were optimized using a Box–Behnken design (BBD) with the aid of Design Expert^®^ (Vers. 13, Stat-Ease, MN, USA) (Stat-Ease Inc., Minneapolis, MN, USA). AgNP concentration (X1), methyl anthranilate solution volume (X2), and microwave power (X3) were chosen as the three most important independent variables (factors) for optimization at three different levels, namely low (−1), medium (0), and high (+1). The chosen design recommended a total of 17 experimental trials, as indicated in Table 1. Responses included an analysis of the particle size (nm) (Y1) and absorbance (Y2) of produced silver nanoparticles. The data were entered into BBD, and then mathematical modelling was conducted to examine the outcomes. The data fitting the chosen quadratic second-order model was examined using ANOVA along with other factors, including the coefficient of correlation (R^2^), adjusted R^2^, anticipated R^2^, and predicted residual sum of squares. The numerical desirability function and graphical optimization technique helped identify the optimal conditions needed for the synthesis of silver nanoparticles.

### 2.4. Characterization of Silver Nanoparticles

#### 2.4.1. UV–Visible Spectroscopy Analysis

There are various ways to describe nanoparticles. The most significant and beneficial phase is the color change brought on by pretreatment. Using a UV-visible spectrophotometer (Electronics India 1372), it was possible to detect the creation of AgNPs by the color changing from green to yellowish-brown to brown [36]. AgNPs were placed in a 10 mm optical quartz cuvette, and their absorbance was measured at wavelengths between 300 and 900 nm. Double-distilled water was utilised as the standard.

#### 2.4.2. Particle Size, PDI and Zeta Potential

After synthesis, the zeta potential, polydispersity index, and mean particle size diameter were all measured in solution form at 25 ± 1 °C in a Malvern zetasizer (Malvern Instrument, Malvern, UK). The quartz cell received 2 mL of silver nanoparticles. Measurements were made three times at a 90-degree angle to the light source [44]. All results were measured in triplicate after samples were diluted with Milli-Q water before analysis.

#### 2.4.3. Entrapment Efficiency (%)

The EE (%) of methyl-anthranilate-loaded AgNPs (MA-AgNPs) was determined using ultracentrifugation-filtration. Before being ultracentrifuged at a rate of 25,000 g/min at a temperature range of 0 to 4 °C, the produced nanoparticles were filtered (Beckman coulter; LE 80). The resulting supernatant was decanted, and the amount of free active ingredient present was then evaluated by diluting with methanol and performing an analysis at 277 nm using a UV-spectrophotometer (UV-1601 model/Shimadzu Corp., from Kyoto, Japan). Entrapment efficiency was calculated using the formula shown below [45].

EE% = Dinitial − Dsupernatant/Dinitial × 100

where, Dinitial is the initial concentration of methyl anthranilate in silver nanoparticles and Dsupernatant is the concentration of methyl anthranilate in the obtained supernatant of the prepared MA-AgNPs formulation.

#### 2.4.4. Transmission Electron Microscopy (TEM)

Transmission electron microscopy (TEM) was used to examine the silver’s dimensions and shape (Jeol, Japan). An accelerating voltage of 80 kV was used to operate the microscope. An aliquot (20 μL) of the silver samples was put on a carbon-coated grid after being initially diluted (1:10) in distilled water. The excess solution was then blotted off the grid with filter paper after the solution had been left on for 1 min. Before imaging, the grids were left to dry for two hours in the grid box. The images were taken at a magnification of 23 kX [46].

#### 2.4.5. Preparation and Evaluation of Silver Nanoparticles Gel

A gel formulation was created in order to keep the optimized MA-loaded AgNPs formulation on the skin for a longer period of time. Using different concentrations (0.5, 1, 1.5, and 2%) of Carbopol 934 as the gelling agent, synthesized AgNPs were made into a gel. A weighed amount of Carbopol was soaked into the water and kept overnight to swell completely. Later, it was gradually added to the colloidal suspension of AgNPs while stirring, and this was followed by the addition of a drop of triethanolamine, which neutralizes the pH and leads to the formation of gel. Following this, a software-assist gel texture analyzer (TA. XT Plus Texture Analyzer, Stable Micro Systems Ltd., Surrey, UK) was used to assess the texture of the formed AgNPs gel. A total of 50 grammes of the developed gel were put into a 100 mL beaker and spread evenly to avoid bubble entrapment. The analytical probe was then twice placed into the beaker’s gel sample at a depth of 15 mm, moving at a speed of 2 mm/s and maintaining a gap of 20 s between compressions [47].

### 2.5. Evaluation of MA-AgNPs Gel

#### 2.5.1. Physical Appearance

The optimized gel formulation’s color, clarity, and consistency were evaluated following the previously reported procedures [48].

#### 2.5.2. pH and Active Ingredient Content

The pH of the gel was measured using a digital pH metre (Mettle Toledo MP 220, Greifensee, Switzerland), and each experiment was carried out in triplicate [49]. A 50 mL volume of phosphate-buffered saline solution (pH 7.4) was added to the gel formulation, which was precisely weighed at roughly 500 mg, to ascertain the active ingredient concentration. The volumetric flask was aggressively whirled in a shaker for two hours [50]. After diluting the material, it was examined with a UV spectrophotometer at 277 nm.

#### 2.5.3. Spreadability

Spreadability was measured by analyzing the gel’s slide and hindrance characteristics. Two glass slides were arranged in an unusual way; one was fastened to a wooden foundation, and the other was suspended from a balance by a hook. A 1 g of gel sample is sandwiched between these two glass slides. The diameter before and after the application of weight was measured (n = 3) after spreading the gel. Additionally, the spreadability was calculated using the formula:

S = W × L/t

where, S is the spreading ability (g/s), W is the given weight (g), L is the distance travelled by the glass slide, and t is the amount of time (s) required to completely divide the slides [51].

#### 2.5.4. Extrudability

In order to evaluate a gel’s extrusion capacity, the amount of gel that was forced out of a tube at a constant weight was evaluated. a 20 g collapsible tube filled with gel and balanced with a one kg fixed weight. The extruded gel was weighed when the cap was removed. Extrudability was determined in terms of grammes per Newton of force applied per minute [52].

#### 2.5.5. In Vitro Active Ingredient-Releasing Activity

To investigate the active ingredient release of methyl anthranilate from optimized MA-AgNPs and MA suspension, an in vitro active ingredient release experiment was conducted using the 1 kDa MWCO dialysis bag method. In a nutshell, 300 mL of phosphate buffer with a pH of 7.4 and 2 mg of MA-AgNPs were added to tightly sealed dialysis film bags that were wrapped around a cylindrical container and agitated at a rate of 100 revolutions per minute. The temperature was kept constant at 32 °C ± 0.5 °C. On a different cylindrical beaker, the methyl anthranilate free suspension was likewise added to the buffer. After aliquots of two millilitres (0.5, 1, 2, 3, 4, 5, 6, 7, 8, 12, and 24 h) were removed and refilled with an equal amount of phosphate buffer to restore sink conditions, spectrophotometric measurements of the samples were performed at lambda max 277 nm [53].

#### 2.5.6. Dermatokinetics Study

For the ex vivo dermatokinetic study, the excised skins of healthy Wistar rats were employed. The methodology and execution of the skin permeation studies and dermatokinetics on rodent skin were duly approved by the Standing Committee of Bioethics Research, Prince Sattam bin Abdulaziz University Al-Kharj, Saudi Arabia (SCBR-020-2023). In this study, the layers of rat skin’s epidermis and dermis were examined to determine active ingredient concentrations. The abdomen rat skin was cut 1 cm^2^, hair was removed, and it washed gently. The rat skin was mounted on a Franz diffusion cell to investigate the skin penetration of MA-AgNPs gel and MA-conventional formulation gel (MA-CFG). For this study, Franz diffusion cell skin samples were taken at 0, 1, 2, 4, and 8 h [54]. Following the collection of skin samples, we washed them for three minutes in 60 °C water, peeled off the layers, cut them into small pieces, and kept them in methanol for 24 h to allow for the thorough extraction of MA. After filtering the recovered MA methanolic extract, several dermatokinetic parameters were estimated, and its MA content was determined using UV spectroscopy [55].

#### 2.5.7. Antioxidant Properties

Oxidative stress is observed to be increased by excessive UV exposure, which causes sunburn, premature ageing, and tumor development [56]. Oxidative stress is characterised by an excessive generation of free radicals and an accumulation of ROS [57]. Antioxidants are commonly employed to prevent the formation of free radicals. MA has the ability to function as an antioxidant [58]. Antioxidant activity was assessed using the accepted 2,2-diphenyl-1-picrylhydrazyl (DPPH) technique. The antioxidant activity of the formulation, i.e., MA-AgNPs, was compared with the standard, i.e., ascorbic acid solution and pure MA solution. The DPPH free radical technique is a popular method for determining antioxidant capabilities. When cooled to room temperature, a DPPH solution, which is typically violet in color, turns colorless because of antioxidants’ capacity to transfer electrons. We treated the sample (0.5 mL) with a DPPH methanolic solution after dissolving it in 3 mL of methanol (0.3 mL). The resulting mixture was left unattended for an hour to allow the process to complete. The sample’s color shift, a sign of its potential as an antioxidant, reflects its ability to donate hydrogen. The control included 3.5 mL of methanol and 0.1 mL of DPPH solution, while the blank had 3.3 mL of methanol and 0.3 mL of sample (0.3 mL). The 517 nm wavelength was used for the spectrophotometric study [59].

$$\%\;Scavenging=\frac{Absorbance\;\left(blank\right)-Absorbance\left(Sample\right)}{Absorbance\left(blank\right)}\times 100$$

#### 2.5.8. Confocal Laser Scanning Microscope (CLSM)

The penetration of hydroalcoholic Rhodamine solution and Rhodamine-loaded AgNPs into rat skin was monitored using confocal laser imaging. For this study, rat skin was prepared and mounted on Franz cells with the stratum corneum facing the donor cell. An AgNP formulation containing rhodamine was put in the donor compartment and applied topically for six hours. The Franz diffusion cell was maintained at 32 ± 0.5 °C with water circulation, and the receptor compartment was filled with 7 mL of phosphate-buffered solution with a pH of 7.4. Skin that had been gently cleaned with distilled water, put on a glass slide, and treated with a drop of humectant (glycerine) after six hours was examined using confocal laser microscopy with excitation (ex) at 540 nm and emission (em) at 630 nm using an argon laser beam. Skin thickness was measured using an optical z-scan using a confocal microscope [44].

#### 2.5.9. Sunburn Protection Factors (SPF) Analysis

The Santos et al. [60] method was used to calculate the SPF. This approach is simple, quick, and uncomplicated. The SPF values for MA, Ag, and MA-AgNPs were determined. Each time, three measurements of the samples’ absorbance were taken in the UV-B wavelength range (290–320 nm), in 5 nm increments. The Mansur equation was used to determine the SPF:

SPF × spectrophotometric = CF × Ʃ EE (λ) × I (λ) × Abs (λ)

where CF stands for the correction factor (=10), EE stands for the erythemal effect spectrum, I stands for the solar intensity spectrum, and Abs stands for the sunscreen absorbance. The values of EE and I are constant [61].

#### 2.5.10. Stability Studies

To support the assertion, a stability investigation was conducted. Three months of stability tests for the MA-AgNPs gel were carried out in accordance with ICH guidelines. To determine its stability, the optimized MA-AgNPs gel was stored in an aluminium-covered container and maintained at a temperature of 25 ± 2 °C and 60 ± 5% RH. Then, once each month, the following parameters were measured: appearance, color, phase separation, clarity, homogeneity, pH, odour, and washability [62].

### 2.6. Statistical Analysis

GraphPad Prism 8.0 (GraphPad Software, San Diego, CA, USA) was used to analyze the study results. The experiments were repeated three times, and the data were summarized using a mean ± SD one-way ANOVA. A *p*-value less than 0.05 (*p* < 0.05) was used as the threshold for statistical significance.

## 3. Results

### 3.1. Optimization of Methyl-Anthranilate-Loaded AgNPs Formulation by Box–Behnken Design

**Figure 1:**
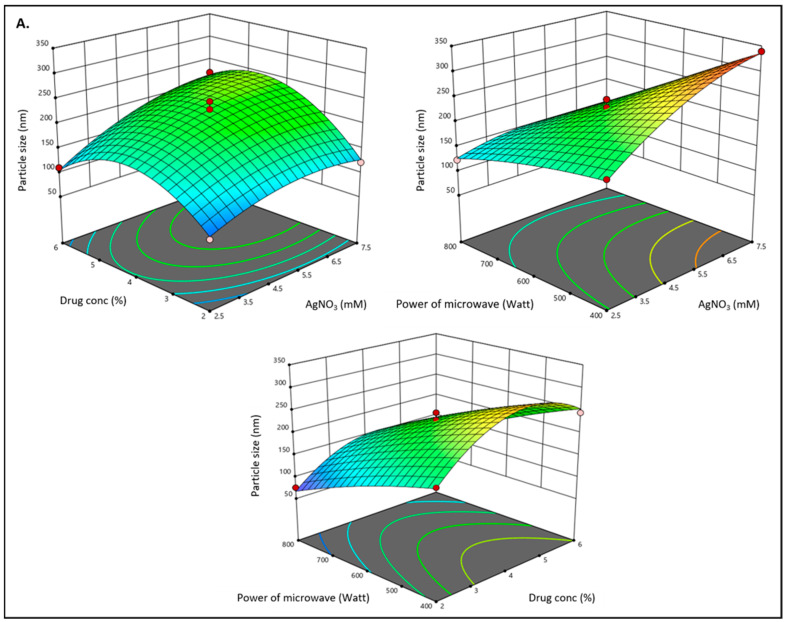

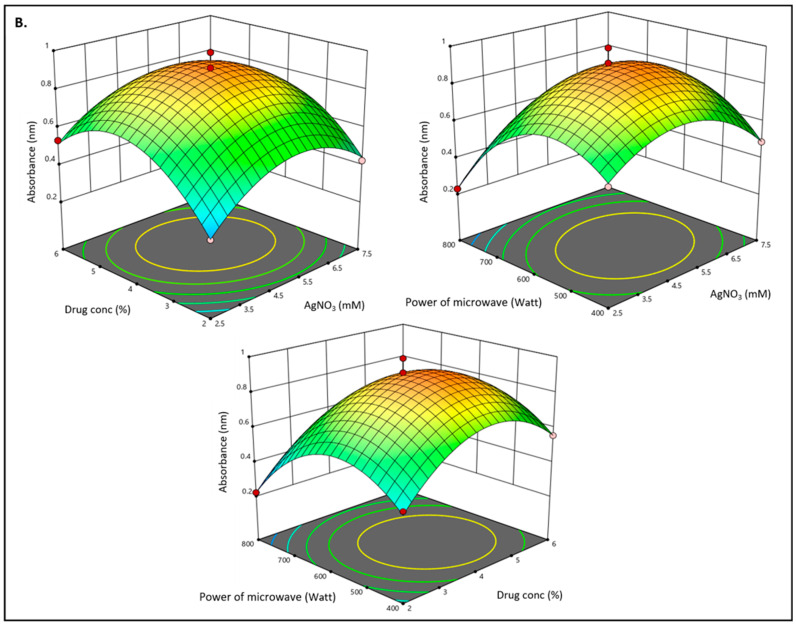
(**A**) 3D surface plot of the relationship between independent variables (AgNO_3_ conc. (X1), drug conc. (X2) and microwave power (X3)) and dependent variables (particle size (Y1), and (**B**) represents a 3D surface plot of the relationship between independent variables (AgNO_3_ conc. (X1), drug conc. (X2) and microwave power (X3)) and dependent variables (absorbance (Y2)).

Three different formulations were developed using the Box–Behnken design after a total of seventeen experimental runs. Table 2 presents the results of these tests. Particle size (Y1) and absorptance (Y2) data ranged from 87.54 nm to 245.89 nm and from 0.229 to 0.991, respectively. The findings of all 17 formulations showed that the quadratic model gave the best fit to the data. Table 3 displays the values for R^2^, standard deviation, and percentage coefficient of variation (CV) for each of the three responses. The link between a number of variables, and particle size (nm), and absorbance is depicted in the three-dimensional graph (Figure 1).

### 3.2. Effect of Independent Variables on Particle Size (Y1)

The developed formulation’s particle size falls between 23.85 to 156.23. The following quadratic equation demonstrated that the AgNO_3_ concentration (mM) had a favourable impact on particle size. AgNO_3_ concentration increases caused the size of the nanoparticle to rise. This is due to the fact that an increase in the AgNO_3_ concentration causes a redshift in the SPR band, which suggests that increasing the Ag+ ions might contribute to the growth of silver nanoparticles and result in a larger particle size [63]. Similar to this, the concentration of methyl anthranilate had a favourable impact on the size of the particle, promoting aggregation of the nanoparticles and resulting in an increase in particle size. However, the microwave’s power had a detrimental effect on the size of the particle, causing it to decrease. Since the surface area to volume ratio rises, this ultimately leads to a reduction in particle size, which increases the overall surface area of the nanoparticle and boosts microwave absorption [36]. As a result, as the microwave’s strength increases, absorption also increases, but particle size decreases.

Particle size (Y1) = +229.69 + 36.37A + 25.39B − 68.30C + 19.14AB − 35.48AC − 8.41BC − 25.22A^2^ −69.06 B^2^ − 8.51C^2^

As the concentration of AgNO_3_ and methyl anthranilate increased, the particle sizes grew larger as they were incorporated into the layers of the nanoparticles. However, according to the equation, the strength of the microwave will still have a negative impact on the size of the particle due to its interaction with the concentrations of AgNO_3_ (AC) and methyl anthranilate (BC). In the analysis, these effects were seen as well-suited.

### 3.3. Effect of Independent Variables on Absorbance (Y2)

According to the proposed equation, it was discovered that the concentration of AgNO_3_ had a positive effect on the absorbance, meaning that as the concentration increased, so did the absorbance value. According to the equation, the absorbance value rises as a result of the methyl anthranilate concentration. However, the microwave’s power had a detrimental impact on the absorbance value.

Absorbance (Y2) = 0.8976 + 0.0358A + 0.0718B − 0.0970C − 0.210AB + 0.0715AC − 0.0010BC − 0.1976A^2^ − 0.2411B^2^ − 0.02686C^2^

### 3.4. Particle Size and PDI of the Optimized Formulation

**Figure 2:**
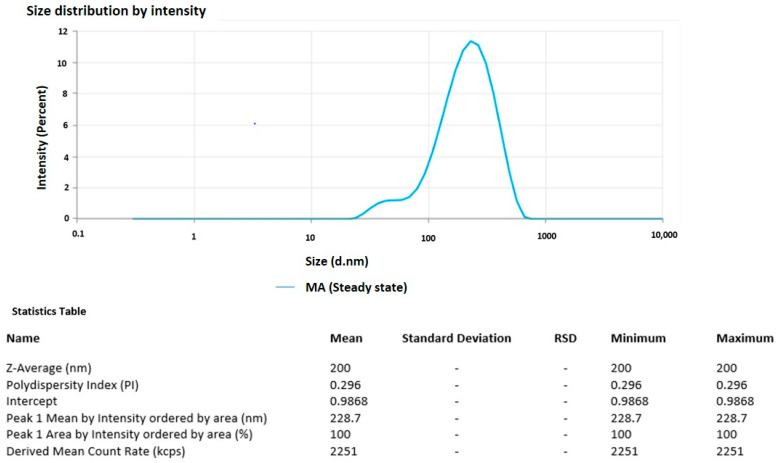
Particle size and PDI of optimized methyl-anthranilate-loaded silver nanoparticles (MA-AgNPs).

The dynamic light scattering method with a nano Zetasizer was used to determine the optimal methyl-anthranilate-loaded AgNPs’ particle size and zeta potential. The optimized MA-AgNPs formulation had a PDI of 0.296 and a particle size of 200 nm. The optimized MA-AgNPs formulation’s particle size is depicted in Figure 2.

### 3.5. Zeta Potential

**Figure 3:**
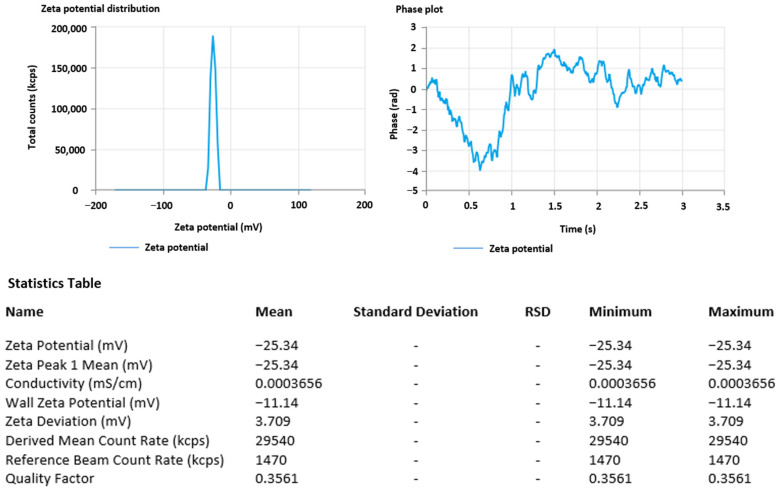
Zeta potential of optimized methyl-anthranilate-loaded silver nanoparticles (MA-AgNPs).

The greater electrostatic repulsion between nanoparticles with higher magnitudes of zeta potential results in more stable nanoparticles. The dramatic peak depicted in Figure 3 of the optimized MA-AgNPs zeta potential was found to be −25.34 mV, validating the formulation’s stability [64].

### 3.6. Percentage Entrapment Efficiency Estimation

The optimized MA-AgNPs formulation’s entrapment efficiency was found to be 87.88%.

### 3.7. Electron Microscopy Imaging

**Figure 4:**
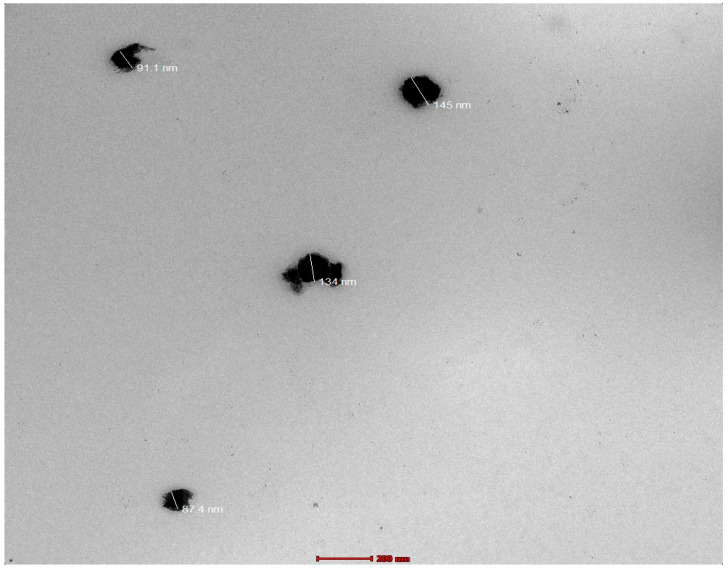
TEM image depicting spherical shapes of optimized methyl-anthranilate-loaded silver nanoparticles (MA-AgNPs) formulation.

In the TEM image of the optimized formulation, methyl-anthranilate-loaded AgNPs were clearly characterized sealed structures with a uniform size distribution and spherical shapes (Figure 4).

### 3.8. Evaluation of MA-AgNPs Gel (MA-AgNPG)

The results of the evaluations of the developed MA-AgNPG’s physical properties are displayed in Table 4. Given that there were no abrasive particles present during the process, the optimized MA-AgNPG was both aesthetically appealing and consistent. The pH of the developed dermal gel is 7.09, which is within the safe range. The MA-AgNPs Gel formulation had an extrudability of 15.19 g and a spreadability of 16.20 g·cm^2^/s, demonstrating that the gel may spread very easily across the skin’s surface, which is beneficial for topical application.

### 3.9. Texture Analysis

**Figure 5:**
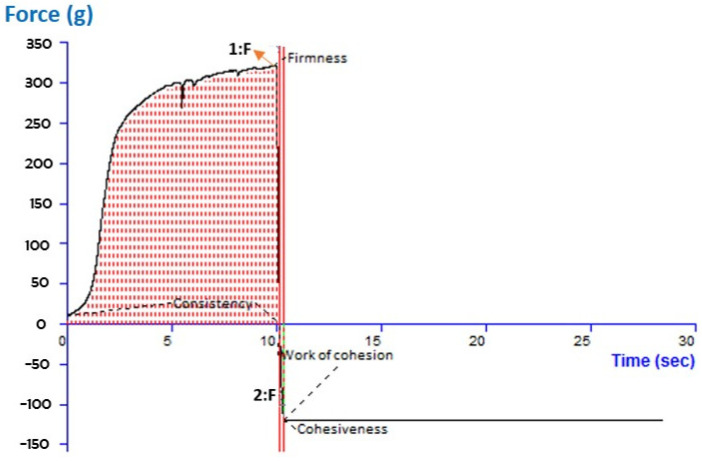
Texture analysis of optimized methyl-anthranilate-loaded silver nanoparticle gel (MA-AgNPG) representing cohesiveness, consistency, firmness, and work of cohesion.

The texture analysis parameters of cohesion, consistency, firmness, and viscosity index were evaluated for the MA-AgNPsG formulation. According to reports, the developed topical gel formulation displayed the following qualities: cohesiveness (−121.44), consistency (2513.16), firmness (322.10), and cohesion work (−16.43). The software’s report on its texture analysis is shown in Figure 5. Firmness is determined by peak or maximum force; the greater the value, the thicker the consistency of the sample. The largest negative force is measured to determine the cohesion or stickiness of the sample. The more negatively skewed the number, the stiffer the sample.

### 3.10. In Vitro Release Study

**Figure 6:**
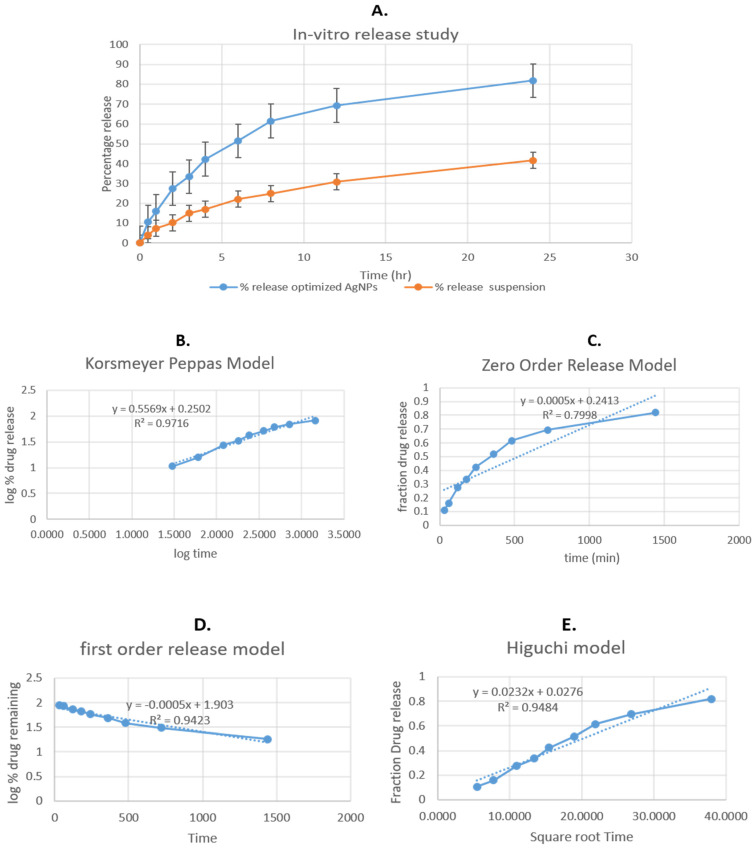
(**A**) Comparative in vitro release profile of methyl anthranilate silver nanoparticles (MA-AgNPs) and methyl anthranilate suspension and in vitro kinetics graphs; (**B**) Korsmeyer–Peppas model, (**C**) first order; (**D**) zero order; and (**E**) Higuchi model of optimized MA-AgNPs formulation.

To evaluate the release of methyl anthranilate, the release behaviour of the prepared, optimized methyl-anthranilate-loaded AgNPs formulation and methyl anthranilate suspension (MA suspension) was studied, as shown in Figure 5. The percentage of active ingredient released by the optimized MA-AgNPs was much higher, at 81.83%, than that released by the MA-suspension, at 41.62%. (Figure 6). The graph shows that there was a burst release at 2 h, which later slowed down. While prolonged slow release increases therapeutic efficacy, initial fast release helps achieve therapeutic concentration. It has shown that our MA-AgNPs formulation results in a controlled release of the entrapped active ingredient over a period of 24 h [36]. Several models, including the zero order, first order, Higuchi, and Korsmeyer-Peppas models, were used to analyze the in vitro release data. To determine the best sequence of releases, we favored the correlation coefficient (R^2^) with the highest value. In the case of optimal MA-AgNPs, the Higuchi model was shown to have the highest correlation coefficient (R^2^ = 0.9484), followed by the first-order model (R^2^ = 0.9423) and the zero-order model (R^2^ = 0.7998). The maximum value of the correlation coefficient was discovered for the optimized MA-AgNPs, establishing Higuchi’s model as the best-fit model. The Korsmeyer–Peppas model revealed that the release of MA from the optimized MA-AgNPs follows non-fickian diffusion; the R^2^ and n values were found to be 0.9716 and 0.556, respectively [36].

### 3.11. Dermatokinetic Study

**Figure 7:**
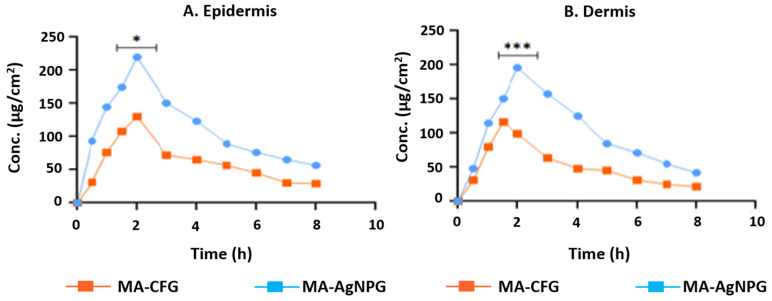
Methyl anthranilate concentration on (**A**) epidermis and (**B**) dermis of methyl-anthranilate-loaded silver nanoparticle gel (MA-AgNPG) and methyl-anthranilate-loaded conventional formulation gel (MA-CFG) (* and *** indicate that the comparison was made between MA-AgNPs Gel and MA-CF gel in the epidermis and dermis).

The distribution of the active ingredient in the rat skin’s dermis and epidermis following the topical delivery of the developed formulation is shown in Figure 7A,B. MA-AgNPs gel significantly increased (*p* < 0.05) MA transport in the epidermal layers when compared to MA-CFG. Additionally, the dermis exhibited a noticeably greater MA AUC (*p* < 0.001) than the MA-CF gel. The numerical information for AUC_0–8 h_, constant skin disposal rate (Ke), C_skin max_, and T_skin max_ is shown in Table 5. The outcomes showed that the MA-AgNPs-gel enhanced active ingredient retention in both layers, contributing to its better efficacy in treating disease caused by UV exposure.

### 3.12. CLSM

**Figure 8:**
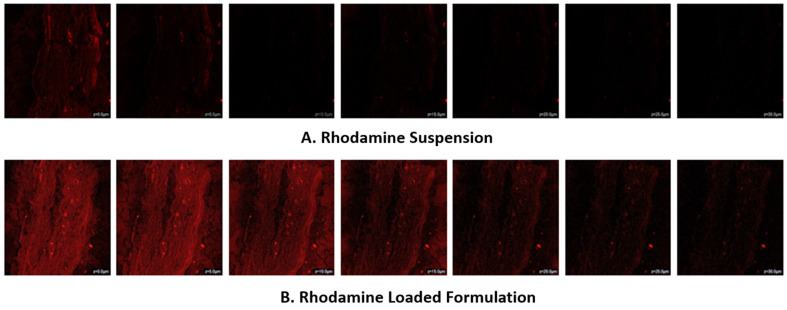
CLSM images of rat skin, (**A**). represents the penetration depth of rhodamine-loaded hydroalacoholic suspension, (**B**). rhodamine-loaded silver nanoparticle formulations (left to right, z = 0 µm, 5 µm, 10 µm, 15 µm, 20 µm, 25 µm, 30 µm).

The amount of depth of Rhodamine B solution permeability across several layers of skin was found in the CLSM investigation. The CLSM results show that the Rhodamine B hydroalcoholic solution was only up to a depth of 5.0 μm in the rat skin (Figure 8A), whereas the CLSM images of Rhodamine B-loaded AgNPs revealed observable fluorescence in the deeper layer of the rat skin with better fluorescence intensity and showed that the Rhodamine B-loaded AgNPs have a strong intensity of fluorescent up to the depth of 35.0 μm (Figure 8B). This indicates improved distribution of AgNPs loaded with rhodamine B in skin strata. This study was approved to assess the level of penetration and topical potency of the optimized AgNPs system as shown by the probe dye’s fluorescence intensity [65].

### 3.13. DPPH Antioxidant Assay

Methyl-anthranilate-loaded silver nanoparticles (MA-AgNPs) were compared to ascorbic acid, a well-established antioxidant benchmark, to determine their level of correlation. Antioxidant activity (% scavenging) was determined to be 98.21 for the reference solution (ascorbic acid), 69.14 for pure MA, and 83.24 for the optimized MA-AgNPs formulation. The data shows that MA-AgNPs had a considerably better effect than pure MA at reducing free radicals. Silver nanoparticles’ enhanced antioxidant action contributed to the elimination of radicles by improving the antioxidant activity of the active component [66]. Resulting in the formulation’s ability to increase the skin’s natural antioxidant capacity and to aid in the neutralization of reactive oxygen species (ROS) caused by environmental variables, including UV radiation [67].

### 3.14. SPF

The sunscreen gel was successfully developed, and it was brown in color and homogeneous with good spreadability. Its effectiveness as sunscreen was calculated as a measure of the SPF, which was determined in comparison to MA gel, AgNPs gel, and the developed MA-AgNPs sunscreen gel. The MA gel, AgNPs, and MA-AgNPs sunscreen gels were found to be 24.9, 9.81, and 35.75, respectively. It was found that the incorporation of MA in AgNPs exhibits an increase in the sun protection factor by 1.5 folds. This could be due to the interaction between MA and the plasmon resonance of silver nanoparticles, which may result in improved UV absorption by loading MA into silver nanoparticles [68], and AgNPs possess a larger surface area, which could enhance the dispersion of the active ingredient, resulting in enhanced protection from UV radiation [38]. Another reason for improved SPF values could be because of their antibacterial capabilities, which can help prevent skin infections and other skin damage that can reduce the efficacy of sunscreen [69,70].

### 3.15. Stability

A study of the AgNPs gel’s physiochemical stability was performed to determine how long it is expected to remain stable after preparation. According to the findings, MA-AgNP gel is the best option. This study shows that MA-AgNPG does not exhibit any phase separation and conforms to all applicable regulatory requirements with respect to active ingredient content, spreadability, and extrudability (Table 6). It was discovered that MA-AgNPG formulations fared best when kept in a cool, dry environment.

## 4. Discussion

In this study, silver nanoparticles were effectively created using microwave assistance. Utilizing a Box–Behnken design with three components at three levels and design-specific software, the formulation was optimized (Version 13). The entrapment effectiveness of the methyl-anthranilate-loaded AgNPs formulation was 87.88%, and the in vitro release was 81.83%, resulting in sub-nonorange particles. Rhodamine B-loaded silver nanoparticles spread more widely across rat skin than the control formulation, according to the CLSM study. Additionally, the DPPH assay was used to establish the optimized methyl-anthranilate-loaded AgNPs formulation’s antioxidant capacity. In comparison to the MA and AgNPs sunscreen gel used alone, the optimized MA-AgNPs sunscreen gel offers a higher SPF rating. The incorporation of AgNPs has synergistically increased the SPF value and made it more efficient against UV-related skin damage. The spreadability and extrudability of the created gel formulation were 16.20 g cm/s and 15.19 gm, respectively, according to the gel characterization research. Additionally, the findings of the texture study demonstrated that the improved AgNPs gel formulation possessed cohesiveness (−121.44), consistency (2513.16), stiffness (322.10), and cohesion work (−16.43) that attest to the manufactured gel of the stable methyl-anthranilate-loaded AgNPs formulation. It was determined that the findings of short-term accelerated stability experiments supported the notion that nanoparticle gel remained stable throughout storage. Although the developed formulation has proven safe and effective for human usage, additional pre-clinical, clinical, and dermatologically verified investigations are required to make sure the new formulation is safe and effective enough to be cleared for public use. The present study’s findings confirmed that the optimized MA-AgNPs formulation gel is an effective approach for topical delivery of methyl anthranilate for the treatment of UV-related skin diseases.

## 5. Conclusions

The microwave-assisted approach proved to be an efficient method for the development of methyl-anthranilate-loaded silver nanoparticles. The MA-AgNPs formulation was optimized using BBD by taking both independent and dependent factors into account. Since the optimized formulation was able to have the optimum particle size, the maximum amount of MA has effectively been entrapped. In vitro active ingredient release studies showed that MA-AgNPs released two folds than MA suspension solutions. The CLSM study on rat skin results demonstrated that the AgNPs formulation containing rhodamine B dye permeated the skin significantly better than hydroalcoholic solutions of the dye, suggesting that AgNPs have enhanced topical protection against UVB-induced damage. In a dermatokinetic study, MA-AgNPs gel was observed to penetrate further into the dermis and epidermis than MA-CFG, proving that the developed gel formulation may be useful for topical applications where greater penetration and longer active ingredient retention are sought. The resulting MA-AgNPs formulation improved the antioxidant activity and also successfully boosted the SPF value for UV protection. These promising results suggest that MA-AgNPs could be used as a topical delivery of MA to treat UVB-induced skin damage. Further pre-clinical/clinical studies and dermatologically tested studies must be carried out in order to establish the possibility of bringing these formulations to market with a low risk/high benefit ratio as compared to a high risk/low benefit ratio in the existing formulation.

## Figures and Tables

**Table 1:** Independent variables (X1, X2 and X3) along with their levels and dependent variables (Y1 and Y2) with their constraints in Box–Behnken design for the preparation of MA-AgNPs nanoparticles.

Design Variables	Low (−1)	Medium (0)	High (+1)
Independent variables			
X_1_ = AgNO_3_ concentration in mM	2.5	5	7.5
X_2_ = methyl anthranilate concentration %	2	4	6
X_3_ = power of microwave in Watt	400	600	800
Dependent variables			
Particle size (Y_1_)	Minimize		
Absorbance (Y_2_)	Maximize		

**Table 2:** Observed values of response in Box–Behnken design (BBD) for optimization of methyl-anthranilate-loaded AgNPs formulations created by Design Expert Software.

	In-Dependent Variables			Responses	
Run	X1 (AgNO_3_ Concentration in mM)	X2 (Methyl Anthranilate Concentration %)	X3 (Power of Microwave in Watt)	Particle Size (nm)	Absorbance
1	5	4	600	200.12	0.991
2	2.5	6	600	110.76	0.534
3	5	6	400	245.61	0.557
4	7.5	4	800	125.43	0.459
5	5	4	600	245.76	0.912
6	5	4	600	245.89	0.893
7	5	4	600	229.81	0.821
8	5	2	400	188.77	0.432
9	5	6	800	98.65	0.342
10	7.5	6	600	221.56	0.548
11	2.5	4	800	123.43	0.229
12	2.5	2	600	87.54	0.328
13	7.5	2	600	121.76	0.426
14	5	4	600	226.87	0.871
15	5	2	800	75.43	0.221
16	7.5	4	400	339.45	0.491
17	2.5	4	400	195.51	0.547

**Table 3:** Summary of arithmetical parameters.

Responses	Analysis	R^2^	Adjusted R^2^	Predicted R^2^	SD	%CV	Model
Particle size (nm)	Polynomial	0.9800	0.9543	0.9160	15.66	8.64	Quadratic Model F Value = 38.16 Adequate Precision = 22.417
Absorbance	Polynomial	0.9817	0.9582	0.9407	0.0501	8.87	Quadratic Model F Value = 41.77 Adequate Precision = 17.6278

Responses Y1 (Particle size) and Y2 (Absorbance).

**Table 4:** Physiochemical characterization of methyl-anthranilate-loaded silver nanoparticles gel (MA-AgNPG).

Homogeneity	Appearance	Washability	Phase separation	Odor
Good	Translucent	Washable	NO	NO
Color	Active ingredient content (%)	pH	Spreadability (g·cm^2^/s)	Extrudability (gm)
Off-white	86.25	7.09	16.20	15.19
Cohesiveness	Consistency (gm·s)	Firmness (gm)	Work of cohesion	
−121.44	2513.16	322.10	−16.43	

**Table 5:** Dermatokinetic parameters for MA-AgNPG and MA-CFG.

Dermatokinetics Parameters	MA-AgNPs-Gel		MACF-Gel	
	Epidermis	Dermis	Epidermis	Dermis
T_skin max_ (h)	2	2	2	2
C_skin max_ (μg/cm^2^)	219.80	195.87	130.03	116.35
AUC_0–8_ (μg/cm^2^h)	903.28	817.35	488.41	411.04
Ke (h^−1^)	0.133	0.111	0.117	0.161

T_skin max_ = time to maximum concentration, Cskin max = maximum concentration, AUC = area under curve, Ke = elimination Rate constant, MA-AgNPs = methyl-anthranilate-loaded silver nanoparticles, and MACF = methyl-anthranilate-loaded conventional formulation.

**Table 6:** Stability studies of the current formulation, which was stored at 25 ± 2 °C/60 ± 5%.

Evaluation Parameters for MA-AgNP Gel	Months			
	Initial	1	2	3
Appearance	No change in appearance			
Phase separation	No phase separation was observed			
Homogeneity	No change in homogeneity			
pH	7.09	7.09	7.07	7.16
Clarity	No change in clarity			
Washability	Washable			
Odor	Good	Good	Good	Good

## Data Availability

Not applicable.
